# Understanding implementation of findings from trial method research: a mixed methods study applying implementation frameworks and behaviour change models

**DOI:** 10.1186/s13063-024-07968-3

**Published:** 2024-02-22

**Authors:** Taylor Coffey, Paula R. Williamson, Katie Gillies, on behalf of the Trials Methodology Research Partnership Working Groups

**Affiliations:** 1 Health Services Research Unit, University of Aberdeen, Health Services Research Unit, Foresterhill, Aberdeen, AB25 2ZD UK; 2 Department of Health Data Science, MRC-NIHR Trials Methodology Research Partnership, University of Liverpool, Liverpool, England

**Keywords:** Trial method research, Implementation science

## Abstract

**Background:**

Trial method research produces recommendations on how to best conduct trials. However, findings are not routinely implemented into practice. To better understand why, we conducted a mixed method study on the challenges of implementing trial method research findings into UK-based clinical trial units.

**Methods:**

Three stages of research were conducted. Firstly, case studies of completed projects that provided methodological recommendations were identified within trial design, conduct, analysis, and reporting. These case studies were used as survey examples to query obstacles and facilitators to implementing method research. Survey participants were experienced trial staff, identified via email invitations to UK clinical trial units. This survey assessed the case studies’ rates of implementation, and demographic characteristics of trial units through the Consolidated Framework for Implementation Research. Further, interviews were conducted with senior members of trial units to explore obstacles and facilitators in more detail. Participants were sampled from trial units that indicated their willingness to participate in interviews following the survey. Interviews, and analysis, were structured via the Capability, Opportunity, Motivation Model of Behaviour. Finally, potential strategies to leverage lessons learned were generated via the Behaviour Change Wheel.

**Results:**

A total of 27 UK trial units responded to the survey. The rates of implementation across the case studies varied, with most trial units implementing recommendations in trial conduct and only few implementing recommendations in reporting. However, most reported implementing recommendations was important but that they lacked the resources to do so. A total of 16 senior members of trial units were interviewed. Several themes were generated from interviews and fell broadly into categories related to the methods recommendations themselves, the trial units, or external factors affecting implementation. Belief statements within themes indicated resources issues and awareness of recommendations as frequent implementation obstacles. Participation in trial networks and recommendations packaged with relevant resources were cited frequently as implementation facilitators. These obstacles and facilitators mirrored results from the survey. Results were mapped, via the Behaviour Change Wheel, to intervention functions likely to change behaviours of obstacles and facilitators identified. These intervention functions were developed into potential solutions to reduce obstacles and enhance facilitators to implementation.

**Conclusions:**

Several key areas affecting implementation of trial method recommendations were identified. Potential methods to enhance facilitators and reduce obstacles are suggested. Future research is needed to refine these methods and assess their feasibility and acceptability.

**Supplementary Information:**

The online version contains supplementary material available at 10.1186/s13063-024-07968-3.

## Background

Clinical trials provide evidence to support decisions about practice in many aspects of healthcare. As well as generating evidence to inform decision making, trials need to, themselves, be informed by evidence in how they are designed, conducted, analysed, and reported to ensure they produce the highest quality outputs [1–3]. This is essential to guarantee not only that trials contribute to evidence-based practice, but that all phases of the trial ‘lifecycle’ also support efforts to minimise research waste by building on best practice for how to design, conduct, analyse, and report trials [1, 2, 4, 5].

Research into how best to design, conduct, analyse, and report clinical trials, known as trial method research [1, 3], has expanded in recent years. For example, a widely studied aspect of trial conduct is recruitment. One project, the Online Resource for Research in Clinical triAls (ORRCA), is an ongoing effort to scope methodological work in recruitment. In their initial publication, the ORRCA team identified 2804 articles, published up to 2015, regarding recruitment [6]. Their most recent update in February 2023 found 4813 eligible papers, an increase of 70% in less than 5 years from the initial publication [6, 7]. As this is just one area of trial methodology, it represents only a fraction of the work being done in this space. With such a large volume of research being generated, coordinated efforts are needed to ensure that learning is shared across research groups to prevent duplication of effort and promote collaboration. There is recognition across the trial method research community that there is significant variability in terms of whether and how the findings from this methodological research influence ‘practice’ with regard to trial design, conduct, analysis, or reporting [3, 8, 9]. Similar to clinical practice, where evidence can fail to be implemented [10, 11], it is critical that the challenges and opportunities to implementing trial method research findings into practice are understood. This understanding will then maximise the potential for this research to improve health by improving the trials themselves.

Barriers to implementation are known to be complex and involve multifactorial influences [12–14]. Whilst this is established for clinical evidence [15], it is also likely to be the case for methodological evidence—yet the specific challenges may be different. Implementation science (and in particular the use of behavioural approaches which are theory-informed) provides a rigorous method for identifying, diagnosing, and developing solutions to target factors with the potential to enhance or impede behaviour change and subsequent integration of those changes [2, 10, 14, 16]. Data generated using these theoretical approaches are likely more reproducible and generalisable than alternatives [2, 16–18]. The potential for lessons from behavioural science to investigate who needs to do what differently, to whom, when, and where, within the context of clinical trials is receiving attention across various stages of the trial lifecycle [2]. The overall aim of this study was to generate evidence for the challenges and opportunities trialists experience with regard to implementing the results from trial method projects that target the design, conduct, analysis, or reporting of trials.

## Methods

### Overall study description

We designed a sequential exploratory mixed methods study with three linked components:

1. *Case studies*: which identified existing examples of trial method research projects with actionable outputs that were believed to influence trial design, conduct, analysis, or reporting practice. “Actionable outputs” were defined broadly as any resource, generated from these projects, that has led to an actual or potential change in the design, conduct, analysis, or reporting of trials.
2. *Survey*: which identified the broad range, and frequency, of challenges and opportunities to the implementation of trial method research. Participants were trialists from across the UK, specifically the Clinical Research Collaboration (UKCRC) Network of Registered Clinical Trials Units (CTUs). The UKCRC was established to “help improve the quality and quantity of available expertise to carry out UK clinical trials.” (https://www.ukcrc.org/research-infrastructure/clinical-trials-units/registered-clinical-trials-units/).
3. *Interviews*: which explored in depth the challenges and opportunities for implementing trial method research from case study examples and general experience in CTU management.

### Theoretical considerations and rationale

It is important when selecting theoretical frameworks, and even more so when combining them within one study, to provide an explicit rationale for the choice of framework(s) [14]. This study utilised a combined theoretical approach, with the Consolidated Framework of Implementation Research (CFIR) [13] guiding the survey development, and the Capability, Motivation, and Opportunity Model of Behaviour (COM-B) [18] guiding the interview guide and analysis. CFIR was designed to synthesise the key elements that underpin implementation efforts [13]. It was selected in this study to guide the survey design because it provided a systematic framework to structure our inquiry. The CFIR is comprehensive in its descriptions of constructs and how they affect implementation across different organisational levels [13]. As the survey was intended to focus more explicitly on the organisational structure of the CTUs, the CFIR possessed the context-specific language and concepts to describe and prioritise our initial findings. The COM-B, in contrast, is broader in its scope as a general theory of behaviour and behaviour change. As implementation efforts largely rely on the adoption and maintenance of new behaviours, or changes to existing ones, behaviour change theory is useful to describe the determinants of behaviour and how they relate to one another [18]. This latter point is particularly relevant for implementation efforts as they are likely to consist of multiple changed behaviours, across different contexts, within an organisation to deliver the ultimate objective of research findings [19]. The COM-B’s capacity to accommodate such complexity outside the prescribed constructs of the CFIR ensured that all relevant factors to implementation are considered [14]. The approaches are further complementary in their conception of the socio-ecological layers within CTUs in which implementation takes place. Again, the CFIR provides the context-specific labels to, and ability to prioritise, these layers, with the COM-B acting as a methodological “safety net” to further describe or categorise findings. And finally, the COM-B is linked to a method of intervention development (and policy functions), known as the Behaviour Change Wheel (BCW). Through the BCW, nine potential categories of interventions are linked to the behavioural domains of the COM-B [18]. This link allows potential solutions to be identified based on the domains found to be most relevant or targetable for the behaviour intended to change.

### Case studies

#### Participants

Members of the Trials Methodology Research Partnership (TMRP) Working Groups (https://www.methodologyhubs.mrc.ac.uk/about/tmrp/) were invited to contribute. Members of these working groups specialise in one or more areas of clinic trial methodology, and all have academic and/or professional interests in improving the quality of trials.

#### Data collection

An email was sent directly to members of the TMRP Working Group co-leads to solicit case studies of trial method implementation projects with actionable outputs. The email included a brief description of the project and aims of the case study selection, followed by two questions. The first question asked for any examples of trial method research that respondents were aware of. Question 2 asked respondents to provide what they believed were the “actionable outputs” (i.e. the resources generated that lead to implementation of findings) of those methods research projects. Examples of potential actionable outputs could include published papers, guidelines or checklists, template documents, or software packages.

#### Data analysis

Responses were collated and reviewed by the research team (TC, PW, KG) for their relevance to the four aspects of design, conduct, analysis, and reporting of trials. These responses were compared with a list of published outputs collected by the HTMR (Network Hubs:: Guidance pack (mrc.ac.uk)) to ensure a wide-reaching range of available trial method research. One case study was chosen for each domain of trial method research through team consensus, resulting in four case studies incorporated into the survey.

### Survey

#### Participants

Directors (or individuals nominated by Directors) of the 52 UKCRC-registered CTUs were invited to participate via email from a central list server independent to the research team.

##### Inclusion and exclusion criteria

Participants were included if they had been involved in any aspect of trial design, delivery, analysis, or reporting within the network of UKCRC-registered CTUs. Any individuals identifying as not reading, writing, or speaking English sufficiently well to participate, or those unable to consent, were excluded.

#### Data collection

The survey was designed, and data collected, via the online survey platform Snap (Version 11). A weblink was distributed to the 52 UK CRC-registered CTUs, along with a description of the study, and a Word document version of the survey (available in Additional file 1: Appendix 1). CTU staff were instructed to distribute this Word version of the survey to members of staff and collate their responses. Collated responses were then entered into the survey at the provided weblink. The survey was designed utilising the Inner Domains of the CFIR [13] to broadly capture participant views on how trial method research informed the design, conduct, analysis, and reporting of trials run through their CTU. It assessed the perceived organizational structure of the CTU and how those demographics influence the adoption of trial method research. It also asked specific questions about each of the case studies selected from the previous phase. Responses consisted of a mixture of single-choice, Likert scales from 1 to 9 (1 being negative valence and 9 being positive valence), and free-text.

#### Data analysis

Examples of trial method research projects suggested by respondents (or research area, e.g., recruitment, if no specific project name was given) were collated and frequency counts for each generated. Frequency counts for the types of actionable outputs from these projects were also calculated. Likert scale responses (ranging from 1 to 9) were analysed through descriptive statistics (mean, standard deviation) to compare responses within and between CTUs, the unit of analysis. Some CFIR domains were assessed by more than one question, and so responses to those questions were averaged to give an overall score for the domain. Scores across all domains for a given site were averaged to give a “general implementation” score. The individual scores on measures of these constructs are presented below using a coloured heatmap to highlight areas of high (green) to low (red) activity and provide easy comparison across and within sites. Additional free-text data were analysed using a directed content analysis approach [20]. Terms and phrases that occurred frequently within this data were collated and then themes summarising barriers and opportunities were generated.

### Interviews

#### Participants

Survey responders indicated their willingness to be contacted for participation in an interview. Emails were sent directly to those who indicated interest in participating.

##### Inclusion and exclusion criteria

Participants were included if they had been involved in any aspect of trial design, delivery, analysis, or reporting within the network of UKCRC-registered CTUs. Any individuals identifying as not reading, writing, or speaking English sufficiently well to participate, or those unable to consent, were excluded.

#### Recruitment and data collection

Interviews were conducted by a trained qualitative researcher (TC) and structured using a theory-informed topic guide. This topic guide (Additional file 2: Appendix 2) was developed using the COM-B Model of Behaviour [18]. Questions prompted interview participants to consider the behavioural influences relevant to implementing findings from trial method research generally and from the selected case studies. Interviews were conducted and recorded through Microsoft Teams. Verbal consent to participate in interviews was obtained and recorded prior to interviews beginning. Recordings were transcribed verbatim by a third party (approved by the University of Aberdeen), de-identified, and checked for accuracy.

#### Data analysis

Data from interviews were imported into NVivo (V12, release 1.6.1) and analysed initially using a theory-based (COM-B) content analysis [20], which allowed data to be coded deductively informed by the domains of the COM-B. This involved highlighting utterances within the transcripts and assigning them to one of the six behavioural sub-domains: “psychological capability”, “physical capability”, “social opportunity”, “physical opportunity”, “reflective motivation”, or “automatic motivation”. The next phase of analysis was inductive, allowing identification of additional themes that may have been outside the COM-B domains but were still deemed relevant to the research question. One author (TC) completed coding independently for all interviews. A second author (KG) reviewed a 10% sample of interviews and coded them independently. Coding was then compared for agreement and any discrepancies resolved. Data were compared and coded through a process of constant comparison to provide a summary of key points that interview participants considered to be important. Interview data were specifically explored for any difficulties reported by trialists with regard to the challenges, opportunities, and potential strategies to facilitate the implementation of findings. These data were collected under “belief statements”, which collected similar statements made across participants under a descriptive heading informed by the statements’ COM-B domain. For instance, similar statements on the availability of resources could be collected under a belief statement, “We do not have enough resources”, representing a barrier within the COM-B domain of “physical opportunity”. Belief statements were then analysed for themes across COM-B domains. These themes were developed as narrative summaries of recurrent experiences, barriers, and facilitators to implementation of methods findings. Themes are presented below with their component COM-B domains indicated within the theme’s title. This thematic framework was reviewed, refined, and agreed by consensus of the research team.

### Identifying potential solutions

Relevant COM-B domains identified during the interviews and agreed by group consensus were mapped to behavioural intervention functions. Mapping of intervention functions was based on instructions within a behavioural intervention guideline known as the Behaviour Change Wheel (BCW) [18]. The BCW describes the intervention functions that are believed to influence the individual domains of the COM-B. For example, a lack of psychological capability could be targeted with the intervention function “Education”, which is defined as “increasing knowledge or understanding” [18]. More than one intervention function is available for each COM-B domain and domains often share one or more intervention functions in common. Utilising the definitions and examples of intervention functions applied to interventions, the research team generated potential solutions based on the available intervention functions targeting the relevant COM-B domains. These solutions were additionally based on the research team’s impressions of targetable belief statements within relevant COM-B domains. For example, if a lack of knowledge was identified (and thus psychological capability) a blanket educational intervention would not necessarily be fit for purpose if only a particular group within an organisation lacked that knowledge whilst others did not. The potential solutions were refined through application of the Affordability, Practicability, Effectiveness and cost-effectiveness, Acceptability, Side-effects and safety, Equity (APEASE) criteria. Application of these criteria to the selection of intervention functions is recommended by the BCW so that research teams can reflect on factors that may limit the relevance and suitability of potential solutions to stakeholders [18].

## Results

### Case studies

Six of 16 Working Group co-leads responded with potential case studies for inclusion. Participants identified a number of trial method research projects, and the project’s outputs, via free-text response to the email prompts. A total of 13 distinct projects were reported by the respondents, primarily in the areas of trial design and analysis, with a particular emphasis on statistical and data collection methods. As a result, case studies for methods research targeting the other two areas of a trial lifecycle, conduct, and reporting, were selected from the list collated by the research team. The four case studies [21–24] were selected to consider the variability of project focus across the four areas of trial method research. The selected case studies are described below in Table 1.

**Table 1:** The four case studies selected

Case study name	Area	Date of publication	Brief description of project objectives	Actionable output(s) – (type of output)
*Developing progression criteria for internal pilot studies* [24]	Design	2017	“This paper outlines the key issues to consider in the optimal development and review of operational progression criteria for RCTs with an internal pilot phase.”	Ten top tips for developing and using progression criteria for internal pilot studies.—(Guidelines/recommendations)
*DAMOCLES* [23]	Conduct	2005	“One main aim of the DAMOCLES study was, therefore, to develop a template for a charter to systematically describe the operating practices and procedures of a DMC.”	“The charter proposed in this paper aims to promote a systematic and transparent approach to the structure and operation of DMCs. […] A worked example of the charter is available on the internet.” – (Template document)
*Statistical analysis plans (SAPS)* [22]	Analysis	2017	“[…] to develop recommendations for a minimum set of items that should be addressed in SAPs for clinical trials, developed with input from statisticians, previous guideline authors, journal editors, regulators, and funders.”	“Recommendations are provided for a minimum set of items that should be addressed and included in SAPs for clinical trials. Examples are provided to illustrate each item, along with an explanation of the rationale and detailed description of the issues to be addressed.” – (Guidelines/recommendations)
*RECAP* [21]	Reporting	2020	“[…] aims to generate participant-centred, evidence-based recommendations for trialists to implement the dissemination of results to trial participants	“Trial teams should develop appropriately resourced plans and consult patient partners and trial participants on how ‘best’ to share key messages with regard to content, mode, and timing. The study findings provide trial teams with clear guidance on the core considerations of the ‘what, how, when and who’ with regard to sharing results summaries.” – (Guidelines/recommendations)

### Survey

#### Site demographics

A total of 27 UK CTUs (Table 2) responded to the survey, just over half of all UK CRC-registered CTUs (*N* = 52). CTUs were primarily in operation from 10 to 20 years (55%) or more than 20 years (30%). The size of CTUs, by staff number, were divided fairly equally between the small (< 50), medium (50–100), and large (100 +) categories. Most sites characterised themselves as moderately (*n* = 12) to highly stable (*n* = 12) in regard to staff turnover.

**Table 2:** Site demographics

*N* = 27		*n*
**Age**	< *5 years*	1
	*5–10 years*	3
	10–20 years	15
	20 + years	8
**Staff numbers**	< 50	10
	50–99	7
	100 +	10
**Stability**	Low (1–3)	3
	Moderate (4–6)	12
	High (7–9)	12

#### Inner domains of the CFIR: culture, implementation climate, networks, and communication

Alongside the structural demographic characteristics described above, we assessed other constructs within the CFIR’s Inner domains. The individual scores on our measures of these constructs are presented in Table 3 below using a coloured heatmap to highlight areas of high to low activity and provide easy comparison across and within sites. Most sites (*n* = 24) achieved general implementation scores between 5 and 7. Typically, scores were reduced due to low ratings for available resources (i.e. money, training, time) within the CTU. Time possessed the lowest individual score, with an average of 3.2 (SD = 1.9). The individual item with the highest average score, 8.2 (SD = 1.3), asked whether relevant findings were believed to be important for the CTU to implement. Finally, available training/education resources were the item with the highest variability across sites, with a standard deviation of 2.2.

**Table 3:** Scores on CFIR domains by site; mean for each question given at end of column; general implementation score (which is a mean of scores across questions) for each site are presented in the final column

Site	Implementation climate							Readiness for implementation				General implementation score (SD)
	**Stability**	**Effective communication**	**Findings are relevant, it is important to implement**	**We clearly communicate the goals of implementing the findings**	**We solicit and incorporate feedback**	**Senior members ask other members for help**	**Staff feel they are integral partners in implementing the findings**	**The CTU has the appropriate amount of:**			**Senior members of the CTU are actively involved**	
								Money	Training/ education	Time		
1	4	8	9	5	5	8	8	3	8	3	6	6.1 (2.2)
2	8	8	9	7	6	7	7	5	7	5	6	6.8 (1.3)
3	6	6	9	7	6	8	6	3	5	4	8	6.2 (1.8)
4	6	8	7	6	5	6	5	5	7	4	5	5.8 (1.2)
5	9	8	7	7	4	7	7	6	4	2	7	6.2 (2.0)
6	5	7	9	6	4	4	6	3	4	1	6	5.0 (2.1)
7	4	6	9	5	2	6	4	4	1	1	6	4.4 (2.4)
8	6	8	9	7	7	7	4	2	7	1	9	6.1 (2.7)
9	7	7	8	6	5	6	6	6	6	6	7	6.4 (0.8)
10	7	7	8	5	4	6	6	7	6	5	6	6.1 (1.1)
11	7	6	9	7	7	7	5	2	4	3	7	5.8 (2.1)
12	7	8	8	7	8	8	7	3	4	4	7	6.5 (1.9)
13	5	6	9	7	5	8	8	5	2	1	0	5.1 (3.0)
14	7	8	9	7	7	7	8	9	9	8	8	7.9 (0.8)
15	7	7	9	7	5	8	5	5	5	4	7	6.3 (1.6)
16	7	7	9	7	6	9	6	1	1	1	7	5.6 (3.1)
17	7	8	9	7	7	8	7	4	7	4	8	6.9 (1.6)
18	6	8	9	9	9	9	9	1	3	1	9	6.6 (3.4)
19	3	4	4	6	3	3	6	4	3	3	7	4.2 (1.5)
20	8	8	8	5	5	8	8	5	7	1	9	6.6 (2.3)
21	3	5	5	7	7	8	7	3	5	2	8	5.5 (2.1)
22	3	8	8	5	6	8	4	6	5	4	8	5.9 (1.9)
23	8	6	6	7	7	7	7	6	6	3	8	6.5 (1.4)
24	5	7	9	9	7	8	7	7	3	2	7	6.5 (2.3)
25	6	7	9	6	5	7	5	3	3	3	7	5.6 (2.0)
26	6	6	9	5	5	5	5	7	9	7	5	6.3 (1.6)
27	6	7	8	6	4	7	6	5	5	2	7	5.7 (1.7)
**Mean**	6.0	7.0	8.2	6.5	5.6	7.0	6.3	4.4	5.0	3.2	6.9	N/A

#### Implementation of example case studies

The two case studies that were the most widely implemented were the DAMOCLES charter and the guidelines for statistical analysis plans. Both case studies were implemented fully by a majority of sites (*n* = 21) with a further minority implementing them at least partially (*n* = 5). The recommendations for internal pilots was fully implemented in some sites (*n* = 8), partially in others (*n* = 9), but was not implemented at all in still others (*n* = 10). The RECAP guidance was not implemented at all in 20 sites, partially in five, and fully in two.

Survey participants reported several key obstacles and facilitators to implementation of the case studies. These factors are summarised, along with the degree of implementation of each case study across the CTUs, in Table 4 below. Two of the most frequently cited factors to enhance or hinder implementation related to the dissemination of findings. The first concerned how findings were packaged for dissemination, with survey respondents noting the utility of templates and write-ups of examples. The second related to the communication of new findings. Respondents mentioned professional networks and conferences as useful in keeping CTU staff up to date on relevant methods research. Workshops, presentations, and other events within those networks also provided these same opportunities with the additional benefit of being tailored to translating findings into practice. A frequently mentioned barrier described potentially inadequate dissemination efforts, as participants cited a lack of capacity to “*horizon scan*” for new findings. Time and funding constraints were described as leading to this lack of capacity. Finally within communication, participants reported that if a member of their CTU had been involved in methods research, it was more likely to be implemented.

**Table 4:** Breakdown of implementation of the four case studies. Frequently mentioned facilitators and obstacles to each case study are presented below the implementation counts. *Potential facilitators as study has not been implemented widely

Total sites (***N*** = 27)	**Progression criteria**	**DAMOCLES**	**Statistical analysis plans**	**RECAP**
				
*Facilitators*	• Funder expectations (*n* = 8) • Best practice (*n* = 4) • Perceived as quality/useful (*n* = 4)	• Clear guidance with useful templates (*n* = 8) • Best practice (*n* = 6) • Standardisation (*n* = 6) • Transparency/defining roles (*n* = 5)	• Standardisation (*n* = 9) • Best practice (*n* = 7) • Clear improvements to practice (*n* = 6) • Perceived as quality/useful (*n* = 5)	• Best practice* (*n* = 5) • Improve consistency in practice* (*n* = 2)
*Obstacles*	• No expertise/not typically involved in area (*n* = 8) • Lack of awareness (*n* = 3)	• Time/resource limitations (*n* = 2)	• Other SOP already in use (*n* = 5) • Time/resource limitations (*n* = 2)	• Lack of awareness (*n* = 13) • Unclear how to implement (*n* = 2)

## Interviews

### Participant characteristics

Sixteen individuals (Table 5) participated in interviews, representing CTUs from across the UK. Participants were primarily directors or other senior members of their respective CTUs. Half of respondents (*n* = 8) had been in these roles for less than 5 years, with a further seven being in their roles from 5 to 10 years. Most (*n* = 11) had been working in trials generally for 20–29 years.

**Table 5:** Summary of interview participant characteristics

	**Participant role category (*N* = 16)**		
	*Director/co-director*	*Head/lead of group*	*Academic*
**Number in role**	7	5	4
**Time in role**			
** < *5 years***	1	3	4
***5–10 years***	5	2	-
** > *10 years***	1	-	-
**Time in trials**			
***10–19 years***	1	2	1
***20–29 years***	5	3	3
***30* + *years***	1	-	-

#### Interview findings

Interviews were conducted remotely and typically lasted 30–45 min. Belief statements were generated under the domains of the COM-B. Those domains were psychological capability, reflective motivation, automatic motivation, physical opportunity, and social opportunity. Cross-domain themes were generated from related belief statements to summarise overall content. Seven themes were identified: “The influence of funders”, “The visibility of findings”, “The relevance and feasibility of findings”, “Perceived value of implementation research”, “Interpersonal communication”, “Existing work commitments”, and “Cultural drivers of implementation”. Themes are presented in detail below with the relevant COM-B domains to which they are linked presented in parentheses. The themes are further organised into the socio-ecological levels for which they are most relevant, i.e. at the level of the CTU (Internal), outside the CTU (External), or to do with the findings themselves (Findings).

##### External factors

Theme 1—The influence of funders (social/physical opportunity and reflective motivation).

Interview participants spoke of the influence of funders as important to what trial method research findings are implemented. These influences were comprised of both the resource implication of funding allocation (physical opportunity) as well as the cultural influence that funders possess (social opportunity). With regard to resource implications, there were restrictions on what implementation-related activities trial staff could perform based on the lack of protected time within their roles that could be allocated to implementation (physical opportunity). Secondly, limitations on time were superseded by requirements set out by funders on which trial method research findings needed to be implemented within their trials. If particular findings were deemed necessary by bodies like the NIHR, CTU staff had no choice but to find time to implement them (reflective motivation). Related to these beliefs was the idea that clear efforts at implementing relevant trial method research findings could signal to funders that the CTU team possessed the skills required to conduct trials, thereby increasing the opportunities for funding through a sort of “competitive edge” (reflective motivation).

“I think the progression criteria, as I said, I think is being driven more by the funders expectations rather than anything else, and then other people go, “Well, if the funder expects to see it, I just have to do it,” so then... they might grumble, basically, but if you’re going to put your grant application in, and you want it to be competitive, this is what we have to do.” – Site 7, director

Theme 2—The visibility of findings (social/physical opportunity and psychological capability).

One of the main barriers cited by interviewees was simply knowing about trial method research findings. Participants described the limits on their own time and capacity in “horizon scanning” for new publications and resources, which was often compounded by the sheer volume of outputs (psychological capability).

“I mean probably the greatest competing demand is being up to speed on what’s coming out that’s new. That’s probably where I would feel that… yes, trying to… I know everyone feels like they don’t have enough time to just read and be aware of the stuff coming out, so that’s… I’m more anxious, and I know others are, that there’s stuff being done that we don’t even know about to try and implement, so in some ways we might almost be repeating the wheel of trying to improve best practice in a topic area, and actually someone’s done loads of work on it.” – Site 3, director.

However, interviewees highlighted several resources as means to close this knowledge gap. Dedicated channels for dissemination of important trial method research findings were one means to stay on top of emerging literature. These could be newsletters, websites, or meetings where part, or all, of the agenda was set aside for updates on findings (physical opportunity). Other resources mentioned included more social opportunities to hear about the latest research, at conferences like the International Clinical Trials Methodology Conference (ICTMC) or network events like training and workshops. These events were also cited as important venues to share lessons learned in implementing trial method research findings or to air general frustrations on the complexities of trial conduct and management (social opportunity). Finally, these networking opportunities were identified by interviewees as potent incubators for collaborations, inspiring new trial method projects or establishing links to assess existing ones. Interviewees reported that the opportunity to be involved in these methods projects worked to also raise awareness of their outputs as well as increasing the perceived relevance of these outputs to CTU staff (psychological capability).

“Again, I think I was very aware of [statistical analysis plans] in my previous role as well, so I’d been along to some of the stats group meetings that the CTU networks have run where this had been discussed before it was published. I think they certainly involved a lot of the CTUs in developing that as well and in canvassing comments that went into the paper. I think potentially that would have been easier for people to implement because we’d had some involvement in the developmental bit as well as it went along.” – Site 22, academic

##### Internal factors

Theme 3—Interpersonal communication (psychological capability, social/physical opportunity, and automatic motivation).

As our participants were senior members of their respective CTUs, they often described aspects of their role and how their efforts mesh with the overall culture of the CTU. A recurrent feature reported by interviewees relating to their role was to be the central figure in communicating the importance of implementation convincingly to their staff and trial sites. This meant they had to advocate for the relevance of trial method research findings to their CTU staff and motivate staff on changing their processes to align with the findings (reflective motivation). This aspect of communication could be more challenging with chief investigators if they were not convinced of the utility of implementation within their own trials, particularly if they anticipated opportunity or resource cost to hosting the research itself or the process changes of implementing findings (social/physical opportunity). Regardless of where it originated, such resistance to change could be frustrating and draining to senior members that were attempting to spearhead implementation efforts (automatic motivation).

“R – Was it ever stressful or frustrating to implement certain things?

P – Yes, I would say it can definitely be. I would be lying if I said no. Because change is always.. there’s always a resistance to change in every institution, so it’s not easy to change things. Yes, it can be frustrating, and it can be painful. Things that help are probably when it’s a requirement and when it’s... whatever you do it goes into your SOPs, and then you say, ‘This is how I have to do it, so this is how we will do it.’ But getting to the step of the institution to recognise it, and the people you’re working with, it can be frustrating because there could be arguments like are hard to argue back like, ‘We don’t have the resources, we don’t have the time. Now is not the moment, we’re...’ so there’s all of these things, but also there’s the effort that it takes to convince people that it’s worthwhile doing the change. It’s definitely... it can be frustrating and disappointing, and it takes a lot of energy.” – Site 21, group lead

However, some broader cultural aspects of the CTU appeared to reduce such frustrations. Participants described that their CTU members were often open to new ideas and that such receptivity facilitated implementation (social opportunity). This openness to change was leveraged through the communication skills of senior staff that were previously mentioned and their ability to solicit opinions and feedback from their staff (psychological capability). Such discussions often took place at internal trainings or meetings that incorporated some focus on implementation efforts for the CTU staff (physical opportunity). These opportunities not only afforded discourse on the practicalities of implementation but also helped to raise general awareness of trial method research findings as well as potential adaptations of findings to better suit the individual requirements of the CTU.

“Yes, I mean at our Trials Unit, I run our monthly trial methodology meetings, so these are predominantly attended by statisticians, so we do focus more on trial methodology that’s more statistical in flavour, but we do always cover the new updates and any key publications we’ve seen. I find that’s a great format for getting people interested and excited in these new methods and distilling them down. Generally, across the unit, we have wider… they’re like two forums, just where everyone gets together, and we tend to have bitesize sessions there where we can distil something. Actually, they’re quite useful because internally, we can distil something new to people but in a bitesize chunk so that people are aware and then can take it further and develop specific… if it’s something quite big, then we can develop working groups to look into it and come to a more solid plan of how we can actually implement it if it seems useful.” – Site 25, academic

Theme 4—Existing work commitments (physical opportunity).

Whilst openness to implementation at the CTU, driven by leadership advocating for its importance, was often present in the interviews, resource restrictions were still an ever-present factor impacting the opportunities for CTU staff to improve practice. Interviewees reported that because any change to be implemented required time and effort to action, mentions of these opportunity costs were reflected universally across our sample. The CTU staff, according to their directive, must prioritise the design of new trials and the delivery of ongoing trials.

“But you know, it’s real, it’s a real challenge and intention to be able to keep your eye on the ball and the many different competing priorities that there are. It does sound like a bit of a weak excuse when you say it out loud. So, our focus is on doing the trials, but of course we should always be trying to have an eye on what is the evidence that it’s underpinning what we do in those trials. We should. But with the best will in the world, it’s writing applications, responding to board comments, getting contracts done once things are funded, getting trials underway. The focus is just constantly on that work of trying to win funding and delivering on what you said you were going to deliver, in amongst all the other business of running a CTU or recruiting staff, managing funding contracts, dealing with our institutions, our universities, our local trusts. All the efforts that go into getting trials underway in terms of writing documents and approvals and recruiting sites, you know?” – Site 10, director

Mitigating these resource restrictions often meant looking to other strategies (mentioned in the next theme) that might allow CTU staff to carve out some capacity towards implementation.

Theme 5—Cultural drivers of implementation (psychological capability, physical opportunity, reflective motivation).

As senior members of their respective CTUs, our participants displayed clear motivations to implement trial method research. They expressed that they would like to see the staff in the CTU improve both the uptake of trial method research findings, as well as generating their own method research. This was part of a larger desire to create a culture within their CTUs that encourages and supports research (reflective motivation).

“I hope that within the Trials Unit, I also create an environment where I’m trying to encourage people to not always work to capacity, so they do have the headroom to go away and explore things and to try things and to develop their own research ideas, so that we can say to people okay. Whether it’s looking at different patient information sheets, whether it’s looking at different recruitment strategies, whether it’s looking at different ways of doing data cleaning across sites, looking at different ways of delivering training to people for data entry because we’ve lots of different ways of delivering training and we still get a very high error rate. I’m sure there are other Trials Units that are doing the same thing, so we should be publishing and sharing that with Trials Units. I’m trying to create that environment.” – Site 1, director

Some potential avenues to promote that development were offered by participants. Firstly, participants were confident in their team’s expertise and ability to either generate or implement trial method research findings. This was evidenced through ongoing work being done within their CTU or discussions with their staff on areas they would like to dedicate time to (psychological capability). An important role for the senior members of staff is then to set out expectations for their teams around how they can leverage their expertise within implementing or generating trial method research findings and for senior members to offer the necessary support for that to happen. One option put forward to facilitate this leveraging of expertise was to provide career development opportunities centred on implementation. This could simply be allocating staff’s time to focus on implementation projects, protecting their time from usual work commitments. A further development opportunity would be appointing so-called “*champions*” within the CTU whose explicit role is to identify trial method research findings and coordinate their implementation (physical opportunity).

“Because sometimes what I think is [...] you need a champion, you need every CTU to implement these things and because every trial or every trials unit is composed of different people, so I would probably champion the SAPs part because I’m the statistician, and I make sure that that goes ahead, but someone else needs to champion the one on the patients, probably. Not necessarily. I would champion for all of these things, but because... I think it's finding these people that are the ones that see the value and then be the drivers of the unit. I think that will probably help. […] But I honestly think the best way is just reaching a champion for each of these areas and reaching out to them and saying, ‘Can you... what do you think of this, and what would you do to implement it in your own unit?’” – Site 21, group lead

##### Factors related to findings

Theme 6—Relevance and feasibility of findings (physical opportunity, reflective motivation, and psychological capability).

Not all findings from trial method research are applicable to all trials and there to all CTUs. For instance, some of our participants mentioned that the progression criteria recommendations were not widely implemented by their CTU staff because they did not often include internal pilots in their trials. So, once the challenges of knowing about trial method research findings are overcome, CTU staff then need to make decisions on what is most relevant to their trial portfolio and what they would like to prioritise implementing (reflective motivation). This prioritisation was dependent on two factors, the CTU staff’s ability to adapt findings to their needs and the implementation resources that findings are packaged with. These factors appeared to be interconnected as sufficient resources to aid implementation, such as training workshops, could reduce the burden of adaptation (physical opportunity). Conversely, staff that perceived their CTU as capable of adaptation could do so even when implementation resources were lacking, such as when trial method research findings are only shared via publication (psychological capability).

“I think that resources that are guidance types widely available, well-advertised, are probably the most... the easiest way. Everything that makes it easier for a person that has this little win of saying, ‘Oh, yes, we’ve probably considered doing things differently,’ anything that minimises that burden in a system I do. For example, with the SAPs, it’s not just the paper and the guidance, but it’s the templates and the little things that you say, ‘Oh, I can start from here, and then if I just use this and this, then the work is so much less […]’ It’s just that thinking of resources that at least create an easy start point for a person that is the right person. I think that would be the best strategy for me, and make them widely available and well-advertised and probably, I don’t know, distribute them, contact the CTUs and say, ‘By the way, here’s a nice resource that you can use if you want to improve this and that.’ I think anything like that could probably be the way I would go around improving the implementation and the uptake because I feel that the goodwill is there.” – Site 21, group lead

Theme 7—Perceived value of implementation (reflective motivation).

Following on from the idea that there is the “*goodwill*” to implement trial method research findings, it was unsurprising that our participants reported believing that implementation research is important. Many believed that uptake of findings had clear benefits to improving the practice of their CTU. Even for those findings of trial method research that were less enthusiastically received, this appeared to be because the CTU staff were already operating at a high standard and that trial method research findings served to simply reassure them of the quality of their practices.

“I guess yes, I would say so, they help enhance them. Thinking about the first one on progression criteria, we didn’t really have any standard in house guidance on that, so actually reaching out and using that was great because we needed something to base it on. Whereas I’d say for the others, with the Damocles ones and the one on SAP guidance, we did already have in house guidelines for SAPs and DMC charters, but these bits of work have helped to inform them. In a way, they help clarify that most of what you are doing is good practice and then some additional things that could be added in.” – Site 25, academic

Alongside the efficiency and quality benefits to the CTU and its practices, participants also described a desire to implement findings from trial method research because of their promise to improve the quality of trials, and the evidence they generate, more broadly. For example, this could be improved efficiency leading to cost-effective trials to free up funding for other research. It could also be participant-centred improvements that have both ethical implications as well as bolstering the public’s trust in the research process. And, most importantly it seemed, improvements across trials would lead to better evidence to base healthcare decisions on. Finally, implementation of findings from trial method research helps to signal that the CTU is dedicated to best practice and is innovative in pursuing those ideals. There was a perception that it can lead to increased reputation amongst peers and the public as well as making the applications from the CTU attractive to funders.

“I think they maybe come under some of the reasons that you said already, but they are incentives to do [implementing trials methods research findings] because we’re all in the business of trying to produce evidence for interventions that are going to make a difference usually in the NHS, not always, but depending what it is that we’re trialling. But ultimately, you know, we’re all in the business of trying to produce evidence that’s going to get used and make a difference to the patients, and if that can happen more quickly, cheaply, more efficiently, trials that are run better with an evidence base underpinning what happens in the trials, then yeah, that’s why we should be doing it. That’s all incentives to do it.” – Site 10, director

### Identifying potential solutions

As stated above in “Interview findings”, the COM-B domains identified were psychological capability, reflective motivation, automatic motivation, physical opportunity, and social opportunity. These five domains map to all nine intervention functions within the BCW. Two, “Restriction” and “Coercion”, were eliminated due to limited practicability and acceptability. Potential solutions were generated that targeted specific aspects of beliefs within our themes. The primary factors identified across themes were distilled into three intervention targets. Those targets were as follows: awareness of trial method research findings, the effort required to implement findings, and the culture around implementing findings. Eight potential interventions were generated which are listed in Table 6.

**Table 6:** Potential solutions mapped to intervention functions of the Behaviour Change Wheel; Belief statements that comprise each target are given in paratheses following the target in the first column

Overarching target (Belief statements comprising)	COM-B domains	Intervention functions	Possible strategies for CTU management/staff
**Awareness of findings** (Dedicated channels for dissemination, like network events and conferences, are helpful ( +); Conferences and network events are a great opportunity to share implementation research and experience ( +); It’s difficult to know what research is out there ( −))	Physical opportunity, Social opportunity, Psychological capability	Education, Enablement Environmental restructuring, Modelling, Training	Conference sessions on new trial method research findings—at both clinical and methods conferences
**Effort required to implement findings** (The resources that findings are packaged with help make them more implementable ( +); We have to prioritise what’s relevant to the trials our CTU delivers ( −); You should be able to adapt findings to your CTU ( −))	Physical opportunity, Psychological capability, Reflective motivation	Education, Enablement, Training	Produce recommendations on how to best package findings to make them easier to implement
**Changes to culture** (Funders drive what we prioritise through shifts in research culture ( ~); Involvement in trial method projects support implementation ( +); Funders drive what we prioritise through funding ( ~)	Social opportunity, Physical opportunity	Enablement, Environmental restructuring	Petition funders to provide dedicated funding to CTUs for implementation efforts
**Multiple targets: one or more of the above three targets**	Physical opportunity, Social opportunity, Reflective motivation, Psychological capability	Enablement, Environmental restructuring, Modelling	Establish/modify internal meetings to focus on implementation (Awareness/Culture)
	Physical opportunity	Enablement, Environmental restructuring	Establish role/group of implementation champions (Awareness/Culture)
	Reflective motivation	Education, Incentivisation, Persuasion	Reward staff for successful integration/adherence to changes in practice (Effort/Culture)
	Physical opportunity, Social opportunity, Psychological capability	Education, Enablement, Environmental restructuring, Training	Funders host findings on website, provide resources/training on implementation into methods practice (Awareness/Culture)
	Physical opportunity, Psychological capability	Education, Enablement, Environmental restructuring, Training	Review of current dissemination channels and their level of engagement (Awareness/Culture)

#### Awareness of trial method research findings

The first proposed intervention is the incorporation of sessions specific to sharing research findings into the agendas of clinical and methodology conferences. These sessions would serve as a dedicated conduit for trialists to share and receive new methods research findings, giving dedicated time and space to do so. The social elements of these sessions would also benefit implementation through less formal opportunities to share feedback and other comments on recommendations that can then be addressed by the associated researchers present.

#### Effort required to implement findings

The second proposed intervention would target the effort required to implement findings. As time is at a premium within CTUs, any pre-emptive efforts on the part of the methods research teams to ensure their recommendations are accessible, translatable, and clearly relevant to CTU staff will assist in those recommendations being implemented. This could include template documents, case studies of implementation, software packages, etc. Any resource beyond the publication of results would seem desirable to CTU staff to assist in their efforts at implementation.

#### Changes to culture

The third potential solution identified would target the cultural changes needed to re-prioritise the directions of CTUs towards implementation of findings. This would proceed mainly through a change in funder attitudes towards the importance of trial method research. Funders would need to provide dedicated funding/time within CTU’s contracts and/or trial grants to allow for the proper conduct and/or implementation of trial method research.

#### Other potential solutions

As many of our reported barriers are interconnected, so too do several of our proposed solutions target multiple barriers/opportunities to improve implementation. Many of these rely primarily on cultural shifts within the CTUs themselves, where existing structures are modified to accommodate implementation efforts. For example, ensuring that CTU meeting agendas incorporate dedicated time towards discussing implementation efforts or for roles to be established/re-structured that focus on championing these efforts.

## Discussion

This paper presents findings from our mixed methods study on the challenges and opportunities to implementing trial method research findings. Exploration of notable trial method research findings generated four cases studies that were used to solicit implementation experiences from trial staff through survey and interviews. The survey data allowed us to identify trends in the adoption of the case studies in a sample of half of the registered CTUs within the UK. Demographic data from participating CTUs demonstrated some similarities in implementation factors that are consistent across sites, such as a lack of resources. More positive similarities were identified as well, such as the shared belief that implementation research is important. Participants volunteered a number of motivators, such as adhering to best practice, or barriers, such as time/resource limitations, that affected their CTU’s implementation of these case studies and trial method research findings more generally. Our interviews with senior CTU staff further explored these motivators and barriers to implementation through a behavioural lens. A range of relevant themes across three socio-ecological levels (Findings, Internal, and External) were identified from our behavioural analysis.

Findings-level factors that affected implementation related to the quality and accessibility of the research and its outputs, and its perceived relevance to the trials undertaken in the CTUs. Trial method research findings that were ‘well-packaged’ (e.g., included templates or easy to follow guidance) were believed to assist in implementation. Findings that had clear benefits to the work done at a CTU, such as streamlining processes, or the outcomes of the trials themselves, such as improving their quality, were more readily implemented. Factors internal to the CTUs included the interpersonal communication of the staff, their existing workloads, and the culture surrounding implementation. Open communication between members of the CTU, spearheaded by senior staff, seemed to increase buy-in from staff on the relevance of trial method research findings. This buy-in would appear essential to motivate staff that are already stretched thin by their commitments to design and deliver trials. Efforts to improve cultural expectations around implementation were seen as a mechanism to create further opportunities for staff to dedicate to adopting findings. These efforts could be restructuring current staff roles or establishing new ones with a greater focus on implementation rather than strictly trial delivery. External factors affecting implementation of trial method research findings were primarily those linked with the expectations of funders and the availability of findings. Funders were said to drive both cultural expectations related to best practice, as well as creating capacity (or not) for CTU staff through provision of funds that could allow dedicated time for implementation efforts. The availability of findings had to do largely with the channels available for dissemination of findings. The more opportunities trialists had to be exposed to findings, the more likely they were to adopt those findings in their respective CTUs.

### Strengths and limitations

Our project has several key strengths. The mixed methods nature of its design allowed for a more complete investigation of implementation factors than either quantitative or qualitative measures alone. The project utilised a combined theoretical approach, taking advantage of the CFIR in survey design and the COM-B in interview design and analysis. The combination of these approaches ensured that our project had the investigative potential to explore the specific implementation factors and general behavioural factors undermining the successful implementation of trial method research. Others have taken a similar epistemological approach in combining the CFIR and COM-B (and the related Theoretical Domains Framework) to investigate challenges in other contexts [14, 25–27].

Our project solicited input from a variety of stakeholders in CTUs across the UK to ensure a diverse perspective on implementation challenges. However, our sample was primarily those with a statistics background, along with the number of responses to identify case studies being relatively low. We attempted to correct for this low response rate and homogeneity of response by agreeing as a team which case studies to include outside those offered by our respondents. However, we cannot say how selection of other case studies may have affected our responses to the surveys and interviews. It may be that particular projects had inherently different challenges to implementation that are not represented here. However, by including general organisational-level factors that may influence implementation, we have identified factors that are likely to be generalisable to a range of implementation efforts. A further bias is one of self-selection. It is possible that the CTUs and members that responded to our invitations are more active in implementing trial method research findings and would thus be more interested in participating in the project. It may also be that those CTUs that face the most challenges did not have the capacity or motivation to respond to our invitation due to the time it would take away from trial delivery. This may help to explain our response rate of about half of the 52 registered CTUs. Responses could have also been limited in our surveys as we asked CTUs to collate their answers. This may have led to unintended desirability effects, with some staff feeling unable to offer honest opinions on their CTU.

### Recommendations for future

This project has identified a number of areas for future efforts in improving the implementation of trial method research findings. The themes described here can provide a starting point for trial method researchers to consider when implementing and/or disseminate findings from method research. This could include creating plans for how the findings will reach the appropriate CTU teams, how to articulate the importance of findings to those teams, or how to best package those findings to make them more readily accessible, and thus implementable, for the CTU teams. Further, it could prompt methods researchers to consider who should be involved in their research and when, potentially incorporating members from different institutions and organisations who would be required to implement any findings and doing so earlier in the process.

Where these obstacles still exist, future research on the implementation of findings can bridge the gap between research and practice. Our approach describes obstacles and facilitators in a standardised language common to behavioural and implementation science. Along with this clearer articulation of what works, for whom, how, why, and when, links to behavioural theory provides a process to design interventions [18, 28]. Although we have identified some preliminary intervention options, future work could produce potential options not accounted for here, but utilising lessons learned from our findings. Further development of these strategies through selection of BCTs targeting one or more of the identified areas for improvement, refined through co-production with stakeholders, would be the next stage of the intervention design process [18, 29]. Finally, assessment of the effectiveness of these interventions in improving the implementation of trial method research findings would be warranted. Additionally, as our project was sampled from UK CTUs, further work could explore the generalisability of these findings to settings outside the UK, particularly where trial units are noticeably different in their organisation.

## Conclusions

We have presented findings exploring the obstacles and facilitators to the implementation of trial method research findings. Challenges facing CTUs at multiple levels, including demands on time and resources, internal organisational structure, and quality of findings, greatly affect their staff’s ability to incorporate findings into their workflow. We have suggested several potential areas to target with further intervention development based on behavioural theory to maximise the potential for change. These strategies, and others, would need to face refinement and the scrutiny of stakeholders, as well as evaluation of their effectiveness. Ultimately, our project highlights the motivation of trial staff to deliver quality trials underpinned by the latest evidence. However, this motivation is hindered by the realities of ongoing trial logistics and the difficulties faced in identifying this evidence. Trial methodologists will need to work closely with CTU staff, funders, and regulatory bodies to set priorities on what needs to be implemented and how to make that more achievable in light of the challenges faced.

### Supplementary Information

**Additional file 1: Appendix 1. **Survey with PIL. Word document version of the survey circulated to CTUs, which includes a PIL section.

**Additional file 2: Appendix 2. **COM-B topic guide. Topic guide used during interviews.

**Additional file 3:** **Domain 1.** Research team and reflexivity.

## Data Availability

The dataset supporting the conclusions of this article is included within the article (and its additional files). Additional data is available upon reasonable request.

## Abbreviations

APEASE: Affordability, Practicability, Effectiveness and cost-effectiveness, Acceptability, Side-effects and safety, Equity
BCT: Behaviour change technique
BCW: Behaviour change wheel
CFIR: Consolidated Framework of Implementation Research
COM-B: Capability, Motivation, and Opportunity Model of Behaviour
CTU: Clinical trial unit
DAMOCLES: DAta MOnitoring Committees: Lessons, Ethics, Statistics
EQUATOR: Enhancing the QUAlity and Transparency Of health Research
HTMR: Hubs for Trial Methodology Research
ICTMC: International Clinical Trials Methodology Conference
MRC: Medical Research Council
NIHR: National Institute for Health and care Research
ORRCA: Online Resource for Research in Clinical triAls
RECAP: REporting Clinical trial results Appropriately to Participants
SAPs: Statistical analysis plans
TMRP: Trials Methodology Research Partnership
UKCRC: UK Clinical Research Collaboration
