# Supplementary material for: Understanding implementation of findings from trial method research: a mixed methods study applying implementation frameworks and behaviour change models
Source: Trials. 2024 Feb 22;25:139. doi: 10.1186/s13063-024-07968-3 (PMC10885447; doi:10.1186/s13063-024-07968-3)
Supplement: Supplementary file 1 — Additional file 1: Appendix 1. Survey with PIL. Word document version of the survey circulated to CTUs, which includes a PIL section. [file 13063_2024_7968_MOESM1_ESM.docx]

Trials methods Research Implementation Project (TRIP)

**Participant information leaflet**

**What is the purpose of this study?**

The survey intends to identify the challenges and opportunities to the implementation of the findings of trial methods research across the UK Clinical Trials Units. To do so, we will ask some general questions about your CTU’s experiences with implementing the findings of trials methods research into practice. We will also ask questions about specific case studies that have been identified through a mapping exercise conducted with the TRIP oversight group. These case studies sit across each of the four core phases of design, conduct, analysis, or reporting of trials.

**Do I have to take part?**

It is your decision about whether or not you wish to take part. If you do agree to take part and then change your mind, you can withdraw at any time without giving a reason however, the data you have provided to that point would still be included in any analysis.

**What happens next?**

If you would like to take part, please click the ‘next’ tab at the bottom of this page which will take you directly to the questionnaire. The questionnaire will take no longer than x minutes to complete. All information which is collected about you during the course of this study will be kept strictly confidential. If you agree to participate in this study, you may be asked to participate in other projects linked to this research.

There will be no extra benefit to you if you do take part in the study but by doing so you will be helping with this research. We do not anticipate there to be any risks associated with participating in this research.

**What will happen to the results of the study?**

We will use the results of this study to help make decisions about future research in this area. The researchers may also report the findings in a scientific journal and at a scientific research meeting. The information that we report would be completely anonymous and would not identify you in any way.

**What ethical and data permissions are in place?**

This study has been reviewed and received favourable opinion by the University of Aberdeen School of Medicine, Medical Sciences and Nutrition’s School Ethics Review Board. All electronic data collected for the purpose of the research study will be confidentially and securely stored on computer servers maintained by the University of Aberdeen. The study team or other individuals from the University of Aberdeen may look at data collected for the study, to check that the study is being carried out correctly and to check the accuracy of the research study. The University of Aberdeen is the controller for this study and is responsible for looking after your information, using it properly and complying with your rights.  You can find more about this at [www.abdn.ac.uk/privacy](http://www.abdn.ac.uk/privacy) or by contacting us at the address below.

**Whom do I contact if I have a concern or a complaint?**

If you have a concern about any aspect of this study, you should ask to speak to the researcher (Taylor Coffey) or Chief Investigator (Dr Katie Gillies) who will do their best to answer your questions [taylor.coffey1@abdn.ac.uk; k.gillies@abdn.ac.uk]. If you remain unhappy and wish to complain formally, you can do this by contacting the Research Governance Team by emailing [researchgovernance@abdn.ac.uk](mailto:researchgovernance@abdn.ac.uk) or by calling 01224 551123.

**Survey Instructions**: Thank you for taking the time to complete this survey. We would ask that you circulate the Word document version that has been included to all relevant members of your CTU and collate their answers to provide a single response from your CTU to these questions via the electronic survey.

Please answer each of the following questions as fully as possible.

Please provide the name of your CTU:

Section 1: General trials methods project experiences

1. Please tell us of any examples of trials methods research that you believe have had a noticeable impact on the design, conduct, analysis or reporting of trials?

2. For those examples, please specify what the actionable outputs of those projects were? “Actionable outputs” refers broadly to any guidance or resource, generated from a project, that aims to support the implementation of the findings of the research.

Section 2: Questions about your CTU and how it utilises the results of trials methods research

1. How long has your CTU been operating (regardless of mergers/name changes)?
   1. <5 years
   2. 5-10 years
   3. 10-20 years
   4. 20+ years
2. How many staff are employed at your CTU (people, not FTE)?
3. Overall, how stable is the staff team at your CTU?

Not stable No opinion Stable

1. There is effective communication between members of our CTU.

Strongly disagree

Strongly agree

Neither agree nor disagree

1. When the findings are relevant to our CTU, it is important that we implement the findings from trials methods research into our CTU

Strongly disagree

Strongly agree

Neither agree nor disagree

1. We clearly communicate the goals of implementing the findings from the projects with CTU staff and sites

Strongly disagree

Strongly agree

Neither agree nor disagree

1. We solicit and incorporate feedback from CTU and site staff on implementing the findings from these projects

Strongly disagree

Strongly agree

Neither agree nor disagree

1. Senior members of the CTU ask other members for help when implementing the findings from these projects and learn from their input

Strongly disagree

Strongly agree

Neither agree nor disagree

1. CTU staff feel they are integral partners in implementing the findings of these methods projects

Strongly disagree

Strongly agree

Neither agree nor disagree

1. The CTU makes time/space to reflect on what is working/not working when we implement findings from these projects

Strongly disagree

Strongly agree

Neither agree nor disagree

1. The CTU has the appropriate resources (including money, training and education, and time) to implement the findings from these projects
   1. Money:

Strongly disagree

Strongly agree

Neither agree nor disagree

Strongly disagree

Strongly agree

Neither agree nor disagree

- 1. Training/education:
  2. Time:

Strongly disagree

Strongly agree

Neither agree nor disagree

1. Senior members of the CTU are actively involved and accountable when implementing the findings from projects

Strongly disagree

Strongly agree

Neither agree nor disagree

Case study 1 (design): Internal pilot studies: developing progression criteria

Citation: Avery KNL, Williamson PR, Gamble C members of the Internal Pilot Trials Workshop supported by the Hubs for Trials Methodology Research, et al. Informing efficient randomised controlled trials: exploration of challenges in developing progression criteria for internal pilot studies. BMJ Open 2017;7:e013537. doi: 10.1136/bmjopen-2016-013537

Link: <https://bmjopen.bmj.com/content/7/2/e013537>

1. Is your CTU aware of this case study?

Unaware

Somewhat aware

Aware

1. Has your CTU implemented the findings from this case study?

Yes No Partially

If yes, what was the motivation(s) for implementing?

If no, what was the barrier(s) to implementing?

1. Do you feel the findings implemented from this case study are better than your CTU’s previous practices? Why or why not?

Case study 2 (conduct): DAMOCLES

Citation: DAMOCLES Study Group, NHS Health Technology Assessment Programme. A proposed charter for clinical trial data monitoring committees: helping them to do their job well. Lancet. 2005 Feb 19-25;365(9460):711-22. doi: 10.1016/S0140-6736(05)17965-3. PMID: 15721478.

Link: <https://www.sciencedirect.com/science/article/pii/S0140673605179653?via%3Dihub>

1. Is your CTU aware of this case study?

Unaware

Somewhat aware

Aware

1. Has your CTU implemented the findings from this case study?

Yes No Partially

If yes, what was the motivation(s) for implementing?

If no, what was the barrier(s) to implementing?

1. Do you feel the findings implemented from this case study are better than your CTU’s previous practices? Why or why not?

Case study 3 (analysis): Guidelines for the Content of Statistical Analysis Plans

Citation: Gamble C, Krishan A, Stocken D, et al. Guidelines for the Content of Statistical Analysis Plans in Clinical Trials. JAMA. 2017;318(23):2337–2343. doi:10.1001/jama.2017.18556

Link: <https://jamanetwork.com/journals/jama/fullarticle/2666509>

1. Is your CTU aware of this case study?

Unaware

Somewhat aware

Aware

1. Has your CTU implemented the findings from this case study?

Yes No Partially

If yes, what was the motivation(s) for implementing?

If no, what was the barrier(s) to implementing?

1. Do you feel the findings implemented from this case study are better than your CTU’s previous practices? Why or why not?

Case study 4 (reporting): RECAP

Citation: Bruhn H, Campbell M, Entwistle V*, et al* What, how, when and who of trial results summaries for trial participants: stakeholder-informed guidance from the RECAP project. *BMJ Open*2022;**12:**e057019. doi: 10.1136/bmjopen-2021-057019

Link: https://bmjopen.bmj.com/content/12/3/e057019

1. Is your CTU aware of this case study?

Unaware

Somewhat aware

Aware

1. Has your CTU implemented the findings from this case study?

Yes No Partially

If yes, what was the motivation(s) for implementing?

If no, what was the barrier(s) to implementing?

1. Do you feel the findings implemented from this case study are better than your CTU’s previous practices? Why or why not?
2. Are there any other challenges to implementing findings that you feel we have not covered that you feel you should mention? Similarly, any things that have made implementation easier?
3. Are there any particular findings from trials methods research that you believe should be implemented more widely? Please provide up to three findings that you believe should be prioritised.

Consent for further contact

This survey is part of a larger project on implementing findings from trials methods research. We will be conducting an interview study to further explore some of the challenges that CTUs face in implementing project outputs. These interviews will be one session of no more than an hour and will take place at a time that is convenient for you and will be held virtually.

Do we have your permission to contact you about participating in these interviews?

Yes Please provide your email address:

No
