# Supplementary material for: Understanding implementation of findings from trial method research: a mixed methods study applying implementation frameworks and behaviour change models
Source: Trials. 2024 Feb 22;25:139. doi: 10.1186/s13063-024-07968-3 (PMC10885447; doi:10.1186/s13063-024-07968-3)
Supplement: Supplementary file 2 — Additional file 2: Appendix 2. COM-B topic guide. Topic guide used during interviews. [file 13063_2024_7968_MOESM2_ESM.docx]

**Behaviour specification**

**Action:** Implementing trials methods research that targets the design, conduct, analysis or reporting of trials

**Actor:** Directors (or nominated individuals) of UKCRC registered Clinical Trial Units (CTUs)

**Context:** 52 UK Clinical Research Collaboration (UKCRC) registered Clinical Trial Units (CTUs)

**Target:** Trials/networks that are managed by the CTUs

**Time:** Within their tenure as CTU directors

*Inform participants that:*

*[Ask participant for assent to record consent conversation]*

*Thank you for agreeing to participate in this interview. The overall aim of this study is to understand what challenges and opportunities trialists experience when implementing the findings of trials methods research that target the design, conduct, analysis or reporting of trials.*

*The specific aim of these interviews is to determine the broad barriers and facilitators to implementation of findings of trial methods research, as perceived by trialists. We will talk about implementation of findings more generally and then go on to discuss specific experiences implementing case studies that your CTU has implemented. I have a number of questions I’m going to ask you - and I’d like you to give them some thought and answer frankly. We are interested in your experiences and views so there are no right or wrong answers.*

*The interview will be audio-recorded and transcribed. These transcripts will be anonymised. All data will be housed on a secure University of Aberdeen server with access limited to members of the research team. You are free to withdraw from participating at any time, although we’d ask if we could keep the data that we have received thus far.*

Do you have any questions for me before we start?

Do I have your consent to proceed with the interview?

1. What is your current job title?
2. How long have you been in your current role?
3. How long have you worked in trials generally?
   1. Has it always been at this CTU?
4. **(PsC)** What is your understanding of “trials methods research” and the types of findings it generates?
5. **(PsC)** Could you tell me of any examples of trials methods research that you believe have had a noticeable impact on the design, conduct, analysis or reporting of trials?
   1. Has your CTU implemented the results of this research?

*In the survey that you completed prior to these interviews, we asked about your CTU’s experience implementing the findings from four particular case studies, one from design, conduct, analysis, and reporting of trials. As a reminder, those are:*

- *Case study 1 (design): Internal pilot studies: developing progression criteria*
- *Case study 2 (conduct): DAMOCLES*
- *Case study 3 (analysis): Guidelines for the Content of Statistical Analysis Plans*
- *Case study 4 (reporting): RECAP*

Your CTU indicated that you had experience implementing:

Case study 1  Case study 3

Case study 2  Case study 4

**Can you confirm those case studies are the ones you have experience with?**

Yes  No

**If no experience with any case studies:**

Since you’ve indicated that your CTU does not have any experience with these case studies, I’m going to present a brief summary of two at random. I would like you to think through some of the potential challenges to implementing these case studies in your CTU.

**If only have experience with one case study:**

Since you’ve indicated that your CTU has experience with one of these case studies, I’m going to present a brief summary of another at random. I would like you to think through some of the potential challenges to implementing this case study in your CTU.

*[Branching alterations in questions for hypothetical implementation]*

1. **(RM)** Do you feel the findings *[that could be]* implemented from these case studies are better than your CTU’s previous practices?
2. **(RM)** Were there *[could there be]* any drawbacks to implementing these case studies?
3. **(RM)** Do you think it was *[could be]* important to implement these case studies?
   1. Do you think it’s generally important to implement findings?
4. **(AM)** Were there *[could there be]* any incentives for you or the CTU to implement findings from these case studies?
   1. Are there any you think would have motivated you?
5. **(AM)** How did *[would]* you feel about implementing these findings?
   1. Was it ever stressful or frustrating?
6. **(PsC)** Were there any competing demands that *[would have]* interfered with implementing findings, for you in particular or the CTU as a whole?
   1. Are those demands still present?
7. **(PsC)** Based on your experiences implementing these case studies and other findings, do you have any suggestions or strategies that you would recommend for implementing findings?
8. **(PhC)** Did [*would*] you have any particular skills that helped successfully implement these case studies?
   1. Have you gained any since that would have been useful/will be useful?
9. **(SO)** Did [*would*] you have support from your colleagues at the CTU when implementing these case studies?
   1. Who were those sources of support?
      1. Was there anyone else (individuals/organisations)?
   2. Would that support be the same or different now?
10. **(PhO)** What resources were [*would be*] helpful to implement these case studies?
    1. What would have been helpful/helpful in the future?

Those are all the questions we have for you.

Is there anything that you feel that we haven’t touched on or you’d like to expand on?

Thank you for your time.
