# Supplementary material for: Understanding implementation of findings from trial method research: a mixed methods study applying implementation frameworks and behaviour change models
Source: Trials. 2024 Feb 22;25:139. doi: 10.1186/s13063-024-07968-3 (PMC10885447; doi:10.1186/s13063-024-07968-3)
Supplement: Supplementary file 3 — Additional file 3: Domain 1. Research team and reflexivity. [file 13063_2024_7968_MOESM3_ESM.docx]

**Domain 1: Research team and reflexivity**

| Personal Characteristics |  |  |  |
| --- | --- | --- | --- |
| 1.  Interviewer/facilitator | | Which author/s conducted the interview or focus group? | TC |
| 2.  Credentials | | What were the researcher's credentials? *E.g. PhD, MD* | TC, KG, PW – PhD |
| 3.  Occupation | | What was their occupation at the time of the study? | TC – Research fellow; KG - MRC Senior Non-Clinical Fellow; PW - Professor of Biostatistics and NIHR Emeritus Senior Investigator Lead |
| 4.  Gender | | Was the researcher male or female? | TC – Male; KG, PW – Female |
| 5.  Experience and training | | What experience or training did the researcher have? | TC – Experience conducting qualitative interviews with trial staff, experience conducting clinical trials |
| Relationship with participants | |  |  |
| 6.  Relationship established | | Was a relationship established prior to study commencement? | Some staff members interviewed were known to TC from professional networks, although no significant relationships were held. KG and PW both have prior relationships with a number of interviewees through professional networks and research but did not participate in the conduct of interviews. |
| 7.  Participant knowledge of the interviewer | | What did the participants know about the researcher? e*.g. personal goals, reasons for doing the research* | All participants had contact with TC while being recruited and were introduced to the objectives of the overall project and the purposes of the interviews. Some participants may have been aware of previous work conducted as a PhD student. |
| 8.  Interviewer characteristics | | What characteristics were reported about the interviewer/facilitator? e.g. *Bias, assumptions, reasons and interests in the research topic* | TC introduced himself, explaining his past experience and interest in the current research topic. |
| **Domain 2: study design** | | |  |
| Theoretical framework | | |  |
| 9.  Methodological orientation and Theory | | What methodological orientation was stated to underpin the study? *e.g. grounded theory, discourse analysis, ethnography, phenomenology, content analysis* | Lines 124-153, 169-173, 176-180, 197-199, 203-217 |
| Participant selection | | |  |
| 10.  Sampling | | How were participants selected? *e.g. purposive, convenience, consecutive, snowball* | Lines 107-123 |
| 11.  Method of approach | | How were participants approached? e*.g. face-to-face, telephone, mail, email* | Lines 154-183 |
| 12.  Sample size | | How many participants were in the study? | Lines 244, 256-257, 302-303 |
| 13.  Non-participation | | How many people refused to participate or dropped out? Reasons? | No participants invited declined to participate or failed to attend the interview. |
| Setting | | |  |
| 14.  Setting of data collection | | Where was the data collected? e*.g. home, clinic, workplace* | Lines 156-157, 164-169, 180-181 |
| 15.  Presence of non-participants | | Was anyone else present besides the participants and researchers? | No |
| 16.  Description of sample | | What are the important characteristics of the sample? *e.g. demographic data, date* | Lines 244-275, 302-307 |
| Data collection | |  |  |
| 17.  Interview guide | | Were questions, prompts, guides provided by the authors? Was it pilot tested? | No |
| 18.  Repeat interviews | | Were repeat interviews carried out? If yes, how many? | N/A |
| 19.  Audio/visual recording | | Did the research use audio or visual recording to collect the data? | Lines 180-181 |
| 20.  Field notes | | Were field notes made during and/or after the interview or focus group? | No |
| 21.  Duration | | What was the duration of the interviews or focus group? | Line 309 |
| 22.  Data saturation | | Was data saturation discussed? | No |
| 23.  Transcripts returned | | Were transcripts returned to participants for comment and/or correction? | N/A |
| **Domain 3: analysis and findings** | |  |  |
| Data analysis | |  |  |
| 24.  Number of data coders | | How many data coders coded the data? | Lines 207-210 |
| 25.  Description of the coding tree | | Did authors provide a description of the coding tree? | N/A |
| 26.  Derivation of themes | | Were themes identified in advance or derived from the data? | Lines 203-220 |
| 27.  Software | | What software, if applicable, was used to manage the data? | Line 203 |
| 28.  Participant checking | | Did participants provide feedback on the findings? | N/A |
| Reporting | |  |  |
| 29.  Quotations presented | | Were participant quotations presented to illustrate the themes / findings? Was each quotation identified? e*.g. participant number* | Yes, Yes by participant ID |
| 30.  Data and findings consistent | | Was there consistency between the data presented and the findings? | Yes |
| 31.  Clarity of major themes | | Were major themes clearly presented in the findings? | Lines 321-553 |
| 32.  Clarity of minor themes | | Is there a description of diverse cases or discussion of minor themes? | Lines 321-553 |
